# Marginal Zinc Deficiency and Environmentally Relevant Concentrations of Arsenic Elicit Combined Effects on the Gut Microbiome

**DOI:** 10.1128/mSphere.00521-18

**Published:** 2018-12-05

**Authors:** Christopher A. Gaulke, John Rolshoven, Carmen P. Wong, Laurie G. Hudson, Emily Ho, Thomas J. Sharpton

**Affiliations:** a Department of Microbiology, Oregon State University, Corvallis, Oregon, USA; b School of Biological and Population Health Sciences, Oregon State University, Corvallis, Oregon, USA; c Department of Pharmaceutical Sciences, University of New Mexico, Albuquerque, New Mexico, USA; d Linus Pauling Institute, Oregon State University, Corvallis, Oregon, USA; e Moore Family Center for Whole Grain Foods, Nutrition and Preventive Health, Oregon State University, Corvallis, Oregon, USA; f Department of Statistics, Oregon State University, Corvallis, Oregon, USA; DOE Joint Genome Institute

**Keywords:** arsenic, gut, microbiome, zinc

## Abstract

Extensive research shows that dietary variation and toxicant exposure impact the gut microbiome, yielding effects on host physiology. However, prior work has mostly considered such exposure-microbiome interactions through the lens of single-factor exposures. In practice, humans exposed to toxicants vary in their dietary nutritional status, and this variation may impact subsequent exposure of the gut microbiome. For example, chronic arsenic exposure affects 200 million people globally and is often comorbid with zinc deficiency. Zinc deficiency can enhance arsenic toxicity, but it remains unknown how zinc status impacts the gut microbiome’s response to arsenic exposure and whether this response links to host toxicity. Using 16S amplicon sequencing, we examined the combinatorial effects of exposure to environmentally relevant concentrations of arsenic on the composition of the microbiome in C57BL/6 mice fed diets varying in zinc concentration. Arsenic exposure and marginal zinc deficiency independently altered microbiome diversity. When combined, their effects on microbiome community structure were amplified. Generalized linear models identified microbial taxa whose relative abundance in the gut was perturbed by zinc deficiency, arsenic, or their interaction. Further, we correlated taxonomic abundances with host DNA damage, adiponectin expression, and plasma zinc concentration to identify taxa that may mediate host physiological responses to arsenic exposure or zinc deficiency. Arsenic exposure and zinc restriction also result in increased DNA damage and decreased plasma zinc. These physiological changes are associated with the relative abundance of several gut taxa. These data indicate that marginal zinc deficiency sensitizes the microbiome to arsenic exposure and that the microbiome associates with some toxicological effects of arsenic.

**IMPORTANCE** Xenobiotic compounds, such as arsenic, have the potential to alter the composition and functioning of the gut microbiome. The gut microbiome may also interact with these compounds to mediate their impact on the host. However, little is known about how dietary variation may reshape how the microbiome responds to xenobiotic exposures or how these modified responses may in turn impact host physiology. Here, we investigated the combinatorial effects of marginal zinc deficiency and physiologically relevant concentrations of arsenic on the microbiome. Both zinc deficiency and arsenic exposure were individually associated with altered microbial diversity and when combined elicited synergistic effects. Microbial abundance also covaried with host physiological changes, indicating that the microbiome may contribute to or be influenced by these pathologies. Collectively, this work demonstrates that dietary zinc intake influences the sensitivity of the microbiome to subsequent arsenic exposure.

## Abstract (precis)

Xenobiotic compounds, such as arsenic, have the potential to alter the composition and functioning of the gut microbiome. The gut microbiome may also interact with these compounds to mediate their impact on the host. However, little is known about how dietary variation may reshape how the microbiome responds to xenobiotic exposures or how these modified responses may in turn impact host physiology. Here, we investigated the combinatorial effects of marginal zinc deficiency and physiologically relevant concentrations of arsenic on the microbiome. Both zinc deficiency and arsenic exposure were individually associated with altered microbial diversity and when combined elicited synergistic effects. Microbial abundance also covaried with host physiological changes, indicating that the microbiome may contribute to or be influenced by these pathologies. Collectively, this work demonstrates that dietary zinc intake influences the sensitivity of the microbiome to subsequent arsenic exposure.

## INTRODUCTION

The human gut microbiome mediates thousands of interactions between their hosts and xenobiotic compounds daily (1). For example, gut microbes can metabolize xenobiotics, modulate the absorption and dissemination of toxicants (2), and alter bioavailability or activity of pharmaceuticals (3). As microbiome composition is highly personalized, the magnitude of these effects may differ across individuals (4, 5). Environmental factors that alter the composition or function of the microbiome, such as dietary variation (6), may then affect individual variation in response to xenobiotic exposure (5). These exposures could also alter the sensitivity of the microbiome to subsequent xenobiotic exposures, potentially driving the microbiome into a dysbiotic state. Given the well-described associations between microbiome variation and disease (7–11), it is important to understand the extent to which environmental parameters may influence the microbiome’s susceptibility to xenobiotics or its ability to mediate or modify the effects thereof. Yet, no study has explored how dietary variation and environmental chemical exposure interact to affect the microbiome.

Nearly 2 billion people worldwide consume insufficient zinc (12). While the impact of various macronutrients (e.g., fat and protein) on the gut microbiome is well described (6, 13, 14), less is known about how dietary micronutrient variation, such as zinc deficiency, impacts the microbiome and how these impacts may influence host health. The studies that have explored the impact of zinc deficiency on the microbiome find that this single dietary micronutrient can significantly affect the microbiome’s composition (15), which follows from the fact that zinc is an essential nutrient for microbial cells. However, these studies tend to focus on extreme zinc deficiencies (i.e., complete lack of dietary zinc [16, 17]); little is known about how moderate insufficiencies, such as the marginal zinc deficiencies that typically arise from inadequate dietary intake of zinc, impact the microbiome.

Many individuals who consume inadequate amounts of zinc also live in regions where the risk of exposure to toxicants, such as arsenic, is high (18–20). Hundreds of millions of people worldwide (21) routinely consume inorganic and organic forms of arsenic in drinking water and food (22, 23). The concentrations of dietary exposure to the more toxic inorganic forms of arsenic vary widely and frequently exceed safety thresholds (10 µg/liter [19, 21, 24]). Chronic exposure to high arsenic concentrations increases the risk of cancer, cardiovascular disorders, and neuropathies (25). At low concentrations—even those nearing safety thresholds—exposure can also negatively impact health, but the severity of these effects varies (26, 27). This interindividual variation may result from personalized susceptibility to arsenic exposure (21). Several genetic and dietary factors affect arsenic susceptibility (21, 22, 28). Among these factors are micronutrient deficiencies, including zinc deficiency. Zinc and arsenic interact with common proteins (29, 30), and zinc deficiency and arsenic exposure yield similar pathologies (31–33). Moreover, restriction of dietary zinc alters the host’s sensitivity to toxicant exposures, including arsenic (28, 34, 35). Despite these observations, it remains unclear how zinc restriction modulates the physiological effects of arsenic exposure.

While we know that dietary zinc can impact arsenic toxicity in the host (28), we do not understand how marginal zinc deficiency affects the microbiome’s response to subsequent arsenic exposure or how any such combinatorial effects on the microbiome relate to host physiology. For example, consumption of a zinc-deficient diet may enrich for microbes lacking traits required to metabolically detoxify or resist arsenic (36). Consequently, gut bacteria may suffer increased sensitivity to arsenic upon exposure, which may magnify the effects of arsenic on the microbiome. It is important to define how zinc and arsenic interact to affect the gut microbiome because such interactions could contribute to the physiological response of dual exposure in the host. For example, if a zinc-deficient gut microbiome is more sensitive to arsenic, then it is possible that its contribution to homeostasis is more likely to break down upon arsenic exposure.

To determine if multifactorial interactions between zinc, arsenic, and the gut microbiome exist, we examined the impact of physiologically relevant levels of zinc deficiency, through marginal zinc deficiency (37), on the response of the microbiome to environmentally relevant levels of arsenic exposure. Given the independent effects of zinc and arsenic on the gut microbiome, we hypothesized that zinc deficiency and exposure to arsenic yield combinatorial effects on the gut microbiome. We found that arsenic exposure had a modest effect on the microbiome of animals fed zinc-adequate (ZA) diets; however, mice fed marginally zinc-deficient (MZD) diets experienced significant shifts in microbiome composition in response to arsenic exposure. Changes in microbial relative abundance were also associated with host physiological responses to zinc restriction and arsenic exposure. Our data indicate that zinc restriction alters the microbiome’s sensitivity to arsenic exposure and that gut microbes are linked to the physiological changes associated with arsenic exposure and dietary zinc deficiency. Considering the associations between the gut microbiome and health, moderate micronutrient deficiencies may have broader health risks than previously appreciated.

## RESULTS

### Arsenic exposure and zinc restriction alter host physiology.

**FIG 1:**
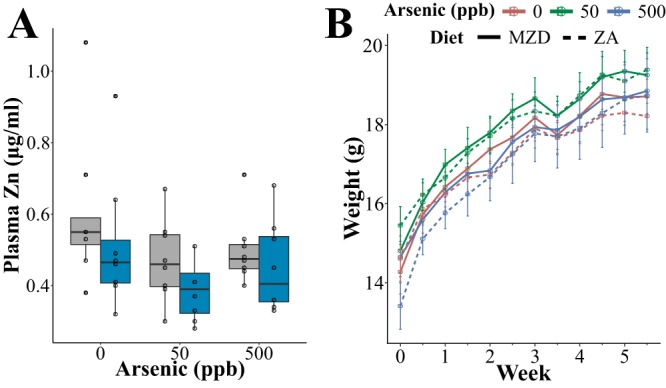
Zinc restriction and arsenic exposure reduce plasma zinc concentrations but not growth of mice. (A) Plasma zinc concentration in animals fed zinc-adequate (ZA) (gray boxes) and marginally zinc-deficient (MZD) diets (blue boxes) and exposed to environmentally relevant concentrations of arsenic. Boxes represent the interquartile range (IQR); the line inside each box represents the median. Upper whiskers on boxes represent the smaller of the maximum value or quartile 3 + (1.5 × IQR). Lower whiskers on boxes represent the larger of the minimum value or quartile 1 − (1.5 × IQR). (B) Weight of mice (g) across the length of the study. Points on each lines indicate the mean weight of animals within a group at a given time point, and whiskers represent the mean ± the standard error of the mean.

To evaluate the effects of arsenic exposure and marginal zinc deficiency on host physiology, both alone and in combination, we exposed C57BL/6 mice fed either ZA or MZD diets to environmentally relevant concentrations of arsenic (50 and 500 ppb) in their drinking water for 6 weeks. After 6 weeks, the plasma zinc concentrations of mice fed MZD diets were significantly lower than those in mice fed ZA diets (*F*_1,42_ = 4.177, *P < *0.05) (Fig. 1A). Plasma zinc levels were also significantly decreased in animals exposed to arsenic (*F*_2,42_ = 3.297, *P < *0.05). However, no interaction between arsenic exposure and zinc restriction was observed (*F*_2,42_ = 0.064, *P = *0.93). Neither arsenic exposure nor diet significantly impacted mouse weight gain (*F*_5,42_ = 0.756, *P = *0.587) (Fig. 1B).

**FIG 2:**
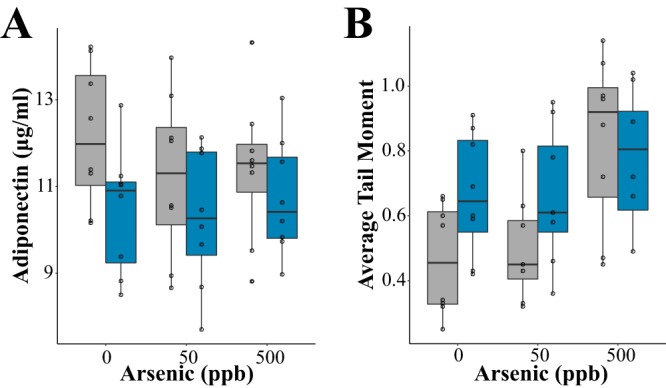
Zinc restriction and arsenic exposure disrupt host physiology. (A) Plasma adiponectin concentrations and (B) comet assay tail moment in zinc-adequate (ZA) and marginally zinc-deficient (MZD) diet-fed animals exposed to arsenic. Boxes represent the interquartile range (IQR), and the line inside each box represents the median. Upper whiskers on boxes represent the smaller of the maximum value or quartile 3 + (1.5 × IQR). Lower whiskers on boxes represent the larger of the minimum value or quartile 1 − (1.5 × IQR).

Animals fed MZD diets had significantly lower plasma adiponectin (*F*_1,42_ = 5.266, *P < *0.05) (Fig. 2A), indicating that oxidative stress may be increased in these animals (38, 39). Arsenic did not significantly impact adiponectin (*F*_2,42_ = 0.494, *P = *0.61). Arsenic exposure (*F*_2,42_ = 6.066, *P < *0.005) and zinc restriction (*F*_1,42_ = 4.357, *P < *0.05) resulted in increased cellular DNA damage (Fig. 2B). These results are consistent with the elevated DNA damage and oxidative stress shown in previous studies (31, 40). Interaction between zinc restriction and arsenic exposure did not significantly contribute to the variation of either adiponectin (*F*_2,42_ = 0.428, *P = *0.65) or DNA damage (*F*_2,42_ = 1.906, *P = *0.16). Together these results suggest that arsenic exposure and zinc deficiency elicit similar physiological effects at environmentally relevant levels of arsenic. However, the combinatorial effects of arsenic exposure and zinc restriction did not significantly impact these host physiological parameters.

### Dietary zinc restriction and exposure to inorganic arsenic diversifies the gut microbiome.

**FIG 3:**
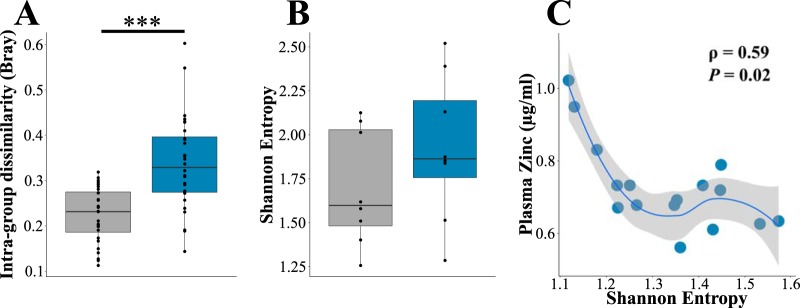
Marginal zinc deficiency alters microbiome diversity. (A) Intragroup Bray-Curtis β-diversity and (B) Shannon entropy of animals fed zinc-adequate (ZA) and marginally zinc-deficient (MZD) diets. Boxes represent the interquartile range (IQR), and the line inside each box represents the median. Upper whiskers on boxes represent the smaller of the maximum value or quartile 3 + (1.5 × IQR). Lower whiskers on boxes represent the larger of the minimum value or quartile 1 − (1.5 × IQR). (C) Scatter plot displaying association between Shannon entropy and plasma zinc concentration. ***, *P < *0.001.

To clarify the effect of dietary zinc deficiency on the gut microbiome, we quantified the differences in gut microbiome biodiversity between MZD- and ZA diet-fed mice. Animals fed zinc-restricted diets displayed higher intragroup β-diversity (Bray-Curtis), a measure of microbiome similarity between members of the same experimental group, than animals fed zinc-adequate diets (*W* = 137, *P < *0.0001) (Fig. 3A). While we observed no differences in Shannon entropy, a measure of microbiome richness and evenness, between MZD- and ZA diet-fed animals (*W* = 21, *P = *0.27) (Fig. 3B), we did find that Shannon entropy negatively correlated with plasma zinc levels in these animals (ρ = −0.59) (Fig. 3C), indicating that the biodiversity of the microbiome may influence plasma zinc concentration and that dietary zinc restriction alters microbiome community composition.

As plasma zinc concentration correlates with diversity, we assessed whether plasma zinc concentration associates with the abundance of specific gut taxa. This analysis revealed few meaningful associations, even at relatively permissive false-discovery rate thresholds (*q* < 0.2). Specifically, using linear regression we found that plasma zinc concentration was positively correlated with the abundance of the genera Shewanella, Rheinheimera, and Bifidobacterium. Unclassified genera of the orders Herpetosiphonales and RF39 also positively associate with plasma zinc. Collectively, these results suggest that zinc restriction causes a stochastic, marginal disturbance to the gut microbiome that increases microbiome diversity, but does not necessarily favor one taxon over another.

**FIG 4:**
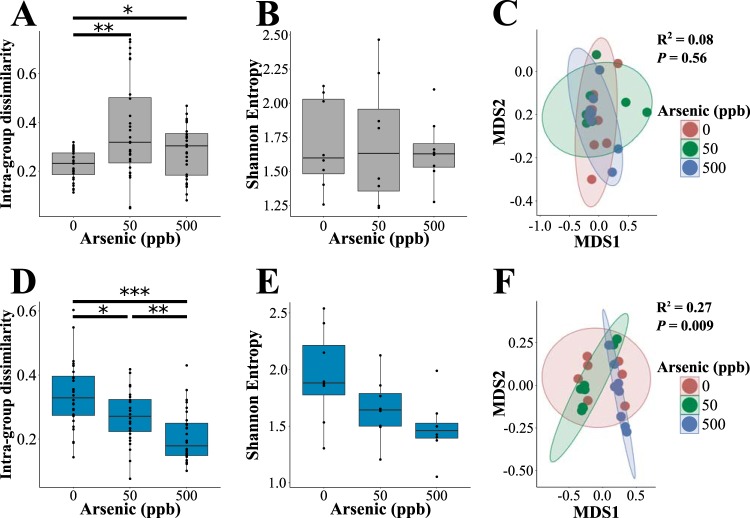
Zinc deficiency sensitizes the gut microbiome to arsenic exposure. (A) Intragroup Bray-Curtis β-diversity, (B) Shannon entropy, and (C) nonmetric multidimensional scaling ordination of β-diversity with adonis *R*^2^ and *P* values for animals fed zinc-adequate (ZA) diets. (D) Intragroup Bray-Curtis β-diversity, (E) Shannon entropy, and (F) nonmetric multidimensional scaling ordination of β-diversity with adonis *R*^2^ and *P* values for animals fed marginally zinc-deficient (MZD) diets. Colored ellipses indicate the 95% confidence interval for each group. For box plots, the boxes represent the interquartile range (IQR), and the line inside each box represents the median. Upper whiskers on boxes represent the smaller of the maximum value or quartile 3 + (1.5 × IQR). Lower whiskers on boxes represent the larger of the minimum value or quartile 1 − (1.5 × IQR). *, *P < *0.05; **, *P < *0.01; ***, *P < *0.001.

We next asked if environmentally relevant levels of arsenic alter microbiome diversity. Mice that were both exposed to 50 or 500 ppb in their drinking water and fed ZA diets displayed elevated intragroup β-diversity (Bray-Curtis) compared to unexposed controls that were fed the same diet [*H*(2) = 10.829, *P < *0.005] (Fig. 4A). Intragroup diversity did not significantly vary between the 50- and 500-ppb groups. Shannon entropy and intergroup β-diversity were not significantly impacted by arsenic exposure in ZA diet-fed animals [*H*(2) = 0.06, *P = *0.97] (Fig. 4B and C). These results suggest that, like zinc, arsenic exposure diversifies the microbiome in a stochastic manner.

### Zinc restriction associates with altered response to arsenic.

To determine if zinc restriction alters the microbiome’s response to arsenic exposure, we quantified the difference in abundance between arsenic-exposed and unexposed animals fed either MZD or ZA diets. Arsenic exposure altered intragroup β-diversity in animals fed ZA [*H*(2) = 21.97, *P < *0.005] (Fig. 4A) and MZD (*P < *0.001) (Fig. 4D) diets. However, the direction of this change differed, depending on the diet: animals fed MZD diets experienced decreased intragroup β-diversity when exposed to arsenic, while ZA diet-fed animals correspondingly increased in diversity. Both MZD and ZA animals had decreased Shannon entropy: however, this reduction was only significant in MZD animals [*H*(2) = 6.305, *P < *0.05] (Fig. 4B and E). While significant differences between individual arsenic doses were not observed in MZD animals (pairwise Wilcoxon test, *P* > 0.1), there was a significant monotonic relationship between Shannon entropy and arsenic concentration (ρ = −0.52, *P = *0.009). No association was detected in ZA animals (ρ = 0, *P = *1). The disparate reaction to arsenic exposure in ZA and MZD animals was consistent at the level of intergroup Bray Curtis β-diversity. The interaction between zinc restriction and arsenic was significantly associated with altered microbial community structure (adonis, *F*_1,47_ = 5.29, *R*^2^ = 0.10, *P < *0.005). The concentrations of arsenic (*F*_1,47_ = 1.41, *R*^2^ = 0.03, *P = *0.21) and dietary zinc (*F*_1,47_ = 1.03, *R*^2^ = 0.02, *P = *0.37) alone did not contribute significantly to the observed variance in intergroup β-diversity. When the diets were considered separately, arsenic exposure was associated with altered microbial community composition in MZD animals (*F*_1,23_ = 6.712, *R*^2^ = 0.23, *P < *0.005) (Fig. 4F) but not ZA animals (*F*_1,23_ = 0.756, *R*^2^ = 0.03, *P = *0.58) (Fig. 4C). Together these data suggest that zinc restriction sensitizes the microbiome to arsenic exposure.

**FIG 5:**
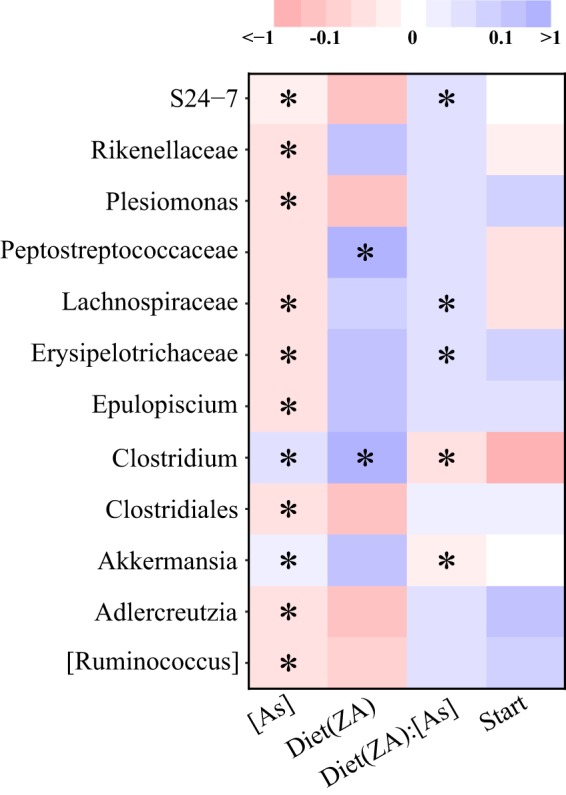
Arsenic exposure and zinc deficiency associate with altered abundance of gut taxa. Shown is a heat map of negative binomial generalized linear model coefficients for the following parameters: arsenic concentration, [As]; zinc status, Diet(ZA); the interaction between zinc status and arsenic concentration, Diet(ZA):[As]; and starting abundance, Start. Red- and blue-colored cells indicate negative and positive slopes, respectively. An asterisk in a cell indicates a *q* value of <0.20.

To further examine microbiome sensitization, we quantified how the abundance of specific gut genera varies as a function of both dietary zinc status and arsenic exposure using robust generalized linear models. The abundance of the genera Adlercreutzia, Ruminococcus, Plesiomonas, and Epulopiscium, as well as unclassified genera within Rikenellaceae, S24-7, Clostridiales, Lachnospiraceae, and Erysipelotrichaceae, was negatively associated with the concentration of arsenic exposure (Fig. 5). Conversely, the abundance of genera Akkermansia and Clostridium was positively associated with arsenic concentration, indicating that the abundance of these taxa increases as arsenic increases. Two genera, an unclassified genus within Peptostreptococcaceae and the genus Clostridium, were significantly elevated in animals fed MZD diets compared to those fed ZA diets (Fig. 5). Significant interaction effects between diet and arsenic exposure on microbial abundance were also observed for the genera Akkermansia and Clostridium, as well as a single unclassified genus within each the families Erysipelotrichaceae, Lachnospiraceae, and S24-7. Interestingly, the abundances of Akkermansia and S24-7 (Fig. 5) disparately respond to arsenic exposure in animals fed MZD and ZA diets, suggesting that zinc restriction may alter the manner in which a microbiome responds to chemical exposure. These analyses indicate that marginal zinc deficiency increases the microbiome’s sensitivity to arsenic exposure and may alter the response of a microbiome to chemical exposure.

### Microbial abundance associates with physiological responses to arsenic exposure.

Arsenic exposure has previously been linked to aberrant DNA damage response and oxidative stress (21, 40). We reasoned that if the microbiome mediates response to arsenic, there should exist associations between the relative abundance of gut taxa and physiological indicators of arsenic exposure, such as DNA damage and oxidative stress. To examine this possibility, we fit linear regressions to quantify the effects of zinc, arsenic, the interaction between zinc and arsenic concentration, and genus relative abundance as the model parameters. We then used an analysis of variance (ANOVA) test to determine if inclusion of taxon relative abundance significantly improved the model fit compared to a reduced model (no genus relative abundance parameter). While we did not observe significant associations (*q* < 0.20) between comet tail moment and the microbiome, serum adiponectin levels were positively associated with the genera Cellvibrio, and Shewanella. Positive associations were also observed between adiponectin and both an unclassified genus within the family Neisseriaceae and an unclassified genus within the class Tenericutes. These findings suggest that some host physiological responses to arsenic exposure associate with the relative abundance of some gut microbiota.

## DISCUSSION

Our study finds that both marginal zinc deficiency and arsenic exposure yield moderate impacts on the composition of the microbiome. However, when zinc deficiency occurs in conjunction with arsenic exposure, these effects on the microbiome magnify. We also observe associations between microbial abundance and indicators of host DNA damage and oxidative stress. Collectively, these results indicate that marginal zinc deficiency sensitizes the microbiome to the impact of environmentally relevant concentrations of arsenic and that these changes to the microbiome are linked to host physiological changes that occur during zinc restriction and arsenic exposure. Moreover, these results highlight that dietary micronutrient status and environmental chemical exposure manifest synergistic effects on the gut microbiome. The finding that dietary micronutrient exposure influences the microbiome’s response to subsequent exposures has implications in almost every field of biomedical research and may help explain some of the variation in microbiome studies in human populations.

Despite the importance of arsenic toxicity and the growing awareness of the microbiome as an agent of health, we understand relatively little about the effects of arsenic exposure on the gut microbiome. Prior research found that exposure to high concentrations of arsenic alters microbial community structure and operation (41). Here, we found that environmentally relevant concentrations of arsenic elicit moderate impacts on microbiome diversity. This finding complements a recent study that also showed that concentrations of arsenic similar to those used here (100 ppb) disrupt microbiome composition and function (42). However, this prior study observed larger effects of arsenic exposure on the microbiome. Chi and colleagues leveraged a longer exposure period in their study (13 weeks), which could account for this variation in effect size. Moreover, facility, strain, or background diet effects could contribute to the differences in magnitude that we observed (43). For example, if the initial microbiome of mice in our facility were enriched for taxa that rapidly detoxified arsenic, then any effects of arsenic exposure in our mice may have been mitigated. Despite the differences in magnitude, both studies highlight the impact of short-term arsenic exposure on the composition of the microbiome. Further study is warranted to determine the minimum exposure length and dose that are sufficient to disrupt the microbiome.

While micronutrient deficiencies are well studied in terms of their effects on health (12), we are only beginning to understand their impact on the gut microbiome. In the case of zinc, prior research demonstrated that severe depletion of zinc (0 to 2.5 µg/g) alters the composition and operation of the gut microbiome (15–17). Our study extends this prior work by demonstrating that marginal dietary zinc deficiency similarly results in a modest, yet significant, restructuring of the gut microbiome. In bacteria, zinc starvation inhibits growth (44), disrupts enzyme activity (45), and increases the expression of zinc transporters (46). Although we did not measure the metabolic outputs of the microbiome during zinc deficiency, it is possible that altered microbial metabolism in MZD animals played a role in the microbiome’s heightened sensitivity to arsenic exposure.

Combining marginal zinc deficiency and arsenic exposure amplified their effects on the gut microbiome. This observation is consistent with the intermediate disturbance hypothesis, which postulates that community diversity maximizes at intermediate levels of ecological disturbance (47). Under this framework, large disturbances, such as frequent antibiotic exposure, reduce diversity as the disturbance selects for a relatively small set of organisms. It is possible that both marginal zinc deficiency and arsenic exposure constitute moderate microbiome-perturbing agents, and therefore their administration results in increased microbiome diversity. However, when applied in combination, their synergistic effect induces a perturbation of far greater magnitude and results in decreasing diversity. If this were the case, then we would expect that micronutrient deficiency would increase susceptibility to many other microbiome-perturbing agents. Moreover, this would also suggest that these deficiencies might lower the exposure threshold needed to perturb the gut microbiome.

Both zinc deficiency and arsenic exposure modulate oxidative stress, inflammation, DNA repair and metabolism (12, 21, 33, 48–51). Correspondingly, our study finds that marginal zinc deficiency and arsenic exposure both independently increased DNA damage and decreased plasma zinc. Cellular DNA damage positively associated with the abundance of the phylum Tenericutes, which prior work links to perturbed gut microbiomes, such as those subject to pathogenic infection (52). Zinc restriction also decreased plasma adiponectin. The family Neisseriaceae, which is depleted in inflamed guts (53), positively associates with adiponectin. These associations suggest that the microbiome may contribute to some of the physiological effects associated with zinc restriction and arsenic exposure.

Collectively, the results of this study bolster the hypothesis that the gut microbiome affects an individual’s physiological response to zinc deficiency and arsenic exposure. A thorough test of this hypothesis ultimately requires demonstrating that the exposure-induced perturbations to the gut microbiome contribute to the physiological responses to those perturbations. Though our study design cannot disentangle cause-and-effect relationships, our results point to potential mechanisms through which zinc deficiency and arsenic exposure may impact the gut microbiome to disrupt physiology. For example, zinc deficiency and arsenic exposure can yield gastrointestinal dysfunctions and elevated intestinal inflammation (54). Our results imply that these exposures could deplete members of the families Rikenellaceae and Lachnospiraceae to consequently contribute to these dysfunctions. Rikenellaceae depletion is associated with impaired mucosal immune function and increased gut inflammation (55). Similarly, Lachnospiraceae contains taxa that produce butyrate, which reduces oxidative stress and inflammation (56), and prior work links their depletion to inflammatory disorders such as Crohn’s disease (57). Correspondingly, MZD animals exposed to arsenic experienced elevated levels of Akkermansia, which contain taxa that induce proinflammatory immune responses in human peripheral blood mononuclear cells (58). Akkermansia has also been shown to exacerbate inflammation during infection with intestinal pathogens (59). These observations specifically point to inflammation as a potential means through which the microbiome mediates the effects of zinc deficiency and arsenic exposure. However, it is unclear if the microbiome plays a role in the proinflammatory immune environment during zinc restriction and arsenic exposure or if the altered microbial abundance was due to altered host physiology in response to marginal zinc deficiency or arsenic. Zinc restriction may also reduce both the host’s and microbiome’s ability to detoxify arsenic exposure. For example, zinc restriction may alter the expression or activity of enzymes involved in the excretion or methylation of arsenic, such as highly conserved arsenic(III) methyltransferase (60). This would increase or prolong the exposure to arsenic, potential enhancing its effects on microbiome and host physiology. It is worth disentangling these relationships in future studies because if the microbiome contributes to the physiological effects of micronutrient deficiency and arsenic exposure, then it may be used as a therapeutic intervention to mitigate these effects. Alternatively, the gut microbiome may be a useful diagnostic for assessing arsenic exposure, though any such diagnostics would need to account for the interacting effects of alternative exposures as documented here.

If the microbiome is important in detoxification of xenobiotics, then any stimuli that alter microbiome composition, function, resistance to perturbation, or resilience may, in turn, alter an individual’s response to subsequent xenobiotic exposures. Thus, based on our prior work and the results of this study, we hypothesize that micronutrient status may have a significant impact on an individual’s response to toxicant exposure such as arsenic. This study demonstrates that coupling zinc deficiency with environmentally relevant exposures to arsenic yields combinatorial effects on the gut microbiome. Moreover, these exposure-induced effects on the gut microbiome correlate with specific changes to physiology. Future work should seek to identify the mechanisms that dictate the microbiome’s sensitivity to zinc deficiency and arsenic exposure as well as the underlying causes of the amplified diversification of the microbiome upon multifactorial exposure. Additional studies are also needed to measure the specific physiological consequences of this diversification and to determine if similar outcomes are observed in humans. Ultimately, the gut microbiome may be proven to be a factor that defines personalized exposure effects, which can help advance microbiome-based preventative therapeutics of exposure.

## MATERIALS AND METHODS

### Animal husbandry, diets, and arsenic exposure.

Forty-eight 4-week-old female C57BL/6 mice were purchased from Jackson Laboratories (Bar Harbor, ME). C57BL/6 mice were selected as they have been used extensively in arsenic toxicity (61), microbiome (41), and zinc deficiency (62) research. Female mice were used in this study to complement our ongoing examination of the effects of low zinc status and arsenic exposure in an at-risk cohort of pregnant Navajo women enrolled in the Navajo Birth Cohort Study (63). To minimize cage-specific effects (64, 65), mice were individually housed in ventilated microisolater cages with BioFresh bedding (BioFresh, Ferndale, WA) and kept in a temperature- and humidity-controlled environment (72°F, 50% humidity, 12-h light cycle). Mice were fed a modified AIN93G diet containing either 30 mg/kg zinc (zinc adequate [ZA]) or 6 mg/kg zinc (marginally zinc deficient [MZD]). We have previously demonstrated that rodents fed this concentration of zinc for 6 weeks exhibit phenotypes consistent with marginal zinc deficiency (48). Diets were formulated with egg whites rather than casein, and zinc was provided as zinc carbonate. Purified ZA and MZD diets were purchased from Research Diets (New Brunswick, NJ). Upon arrival, mice were acclimated to ZA diet for 2 days and then randomly assigned to one of six groups (*n* = 8 mice/group): ZA with 0, 50, or 500 ppb arsenic or MZD with 0, 50, or 500 ppb arsenic. Food and drinking water containing 0, 50, or 500 ppb sodium arsenite were provided *ad libitum* for 6 weeks, with fresh water being provided weekly. These concentrations were chosen because 50 ppb arsenic was the limit set by the Environmental Protection Agency for drinking water up until 2001. Furthermore, arsenic can still be found at 50 to 500 ppb in various groundwater sources around the world (66–68). Food intake and body weights of all mice were monitored twice weekly throughout the study (see Table S1 in the supplemental material). Differences in weight between groups were measured using analysis of variance (ANOVA) and a Tukey’s honestly significant difference *post hoc* test (R stats v3.3.2). Mice were euthanized by CO_2_ asphyxiation at the termination of the experiments, and plasma and tissues were collected. The animal protocol was approved by the Oregon State University Institutional Laboratory Animal Care and Use Committee.

10.1128/mSphere.00521-18.1 TABLE S1 Longitudinal profiling of mouse weight. Biweekly weights (g) of mice fed zinc-adequate (ZA) and marginally zinc-deficient (MZD) diets and exposed to various concentrations of arsenic (0 to 500 ppb). Download Table S1, XLSX file, 0.1 MB. Copyright © 2018 Gaulke et al. 2018 Gaulke et al. This content is distributed under the terms of the Creative Commons Attribution 4.0 International license.

### Plasma zinc and adiponectin measurement.

Plasma zinc concentrations were determined using inductively coupled plasma-optical emission spectrometry (ICP-OES) as previously described (62). Briefly, 50-µl plasma samples were digested in 0.5 ml ultrapure nitric acid and incubated overnight. Incubated samples were diluted with Chelex-treated nanopure water to a final concentration of 10% (vol/vol) nitric acid, centrifuged, and analyzed using the Prodigy high-dispersion ICP-OES instrument (Teledyne Leeman Labs, Hudson, NH) against known standards. ICP-OES analyses were done at the W. M. Keck Collaboratory for Plasma Spectrometry (Oregon State University, Corvallis, OR). Plasma adiponectin was measured using a mouse adiponectin/Acrp30 Quantikine enzyme-linked immunosorbent assay (ELISA) kit (R&D Systems, Minneapolis, MN) per the manufacturer’s protocol. Adiponectin was selected as its concentration has been demonstrated to associate with the zinc concentration (69). In addition, adiponectin levels are significantly reduced in animals exposed to high levels (e.g., 50 ppm) of arsenic (70). However, it was unclear if the impact of varied zinc and arsenic concentration had a synergistic effect on adiponectin levels. Two-way ANOVAs were used to determine if plasma zinc and adiponectin expression significantly varied as a function of arsenic exposure or dietary zinc status.

### Comet assay.

Whole-blood samples were collected at the 5-week time point from the submandibular veins using 4-mm Goldenrod animal lancets (Braintree Scientific, Braintree, MA). DNA damage in peripheral blood cells was determined by alkali single-cell gel electrophoresis (comet assay) as previously described (71). Briefly, whole blood cells were encapsulated in 0.5% low-melting-point agarose and mounted on comet slides (Trevigen, Gaithersburg, MD). Cells were lysed for 1 h to overnight in Trevigen lysis solution. DNA was denatured in alkali electrophoresis buffer (0.3 M NaOH, 1 mM EDTA) for 30 min prior to electrophoresis (25 V, 300 mA, 30 min). Slides were rinsed in water, and dried. DNA was stained using SYBR Gold nucleic acid stain (Thermo Fisher Scientific, Waltham, MA) and visualized using a fluorescence microscope. Images were captured and analyzed using Comet Assay IVTM (Perceptive Instruments, Bury St Edmunds, United Kingdom). A minimum of 100 cells were scored from each mouse, and average tail moment from the individual mouse was reported. A two-way ANOVA quantified variance across groups.

### Microbiome sequencing and bioinformatic analysis.

Mouse fecal pellets were collected from individually housed mice at weeks 0 and 5 and stored at −80˚C until processing. Microbial DNA was extracted using the Powersoil DNA extraction kit (Qiagen, Fredrick, MD) according to the manufacturer’s protocol, with the addition of a 10-min incubation at 65˚C prior to bead beating. The V4 region of 16S rRNA gene was amplified and purified as previously described (72, 73) and sequenced on an Illumina MiSeq using the MiSeq v2 2- × 250-bp reagent kit (Illumina, San Diego, CA).

Microbial sequences were quality filtered and trimmed using the split_libraries_fastq.py script in QIIME (v1.8.0) (74). Quality-filtered (*q* > 20) and trimmed reads were subjected to open reference clustering using the UCLUST algorithm (75) against the Greengenes (version 13_8) 97% operational taxonomic unit (OTU) database (76). Chimeric sequences were identified using ChimeraSlayer and filtered from the OTU table. Microbiome diversity was analyzed using the QIIME script core_diversity_analysis.py and R.

Associations between microbiome diversity and host physiological parameters were determined using permutational multivariate analysis of variance (PERMANOVA; R vegan v2.4.6). Spearman rank sum tests quantified the association between Shannon entropy and plasma zinc concentration. Data were visualized using R and ggplot2 v2.2.1 (77).

### Regression analyses.

Associations between microbial abundance, diet, and arsenic exposure concentration were calculated using negative binomial generalized linear models with a log link function (R, MASS v7.3.48 [78]). A single model was fit for each genus:

$$\text{genus}\;\text{5-wk}\;\text{abundance}=\beta_{0}+\beta_{\text{1}}\left(\text{genus}\;0\text{-wk}\;\text{abundance}\right)+\beta_{\text{2}}\left(\text{diet}\right)+\beta_{\text{3}}\left(\text{arsenic}\;\text{concn}\right)+\beta_{\text{4}}\left(\text{diet}:\text{arsenic}\;\text{concn}\right)+\varepsilon$$

The parameter “diet:arsenic concn” represents the interaction between zinc status and arsenic concentration. The initial abundance (week 0) of the taxa was included in each model to account for potential variance contributed by the starting abundance. The false-discovery rate was controlled using the qvalue package in R (qvalue v2.6.0 [79]).

To quantify relationships between host physiological parameters and microbial taxonomic abundances, we built two linear models (R stats). Model 1 quantified the effect of the interaction between diet and arsenic concentration. Model 2 quantified the effect of the interaction between diet and arsenic concentration with an additional parameter of microbial relative abundance. Specifically the model formulas were as follows:

$$\text{model}\;\text{1}\;\text{:}\;\text{physiological}\;\text{parameter}=\beta_{0}+\beta_{\text{1}}\left(\text{diet}\right)+\beta_{2}\left(\text{arsenic}\;\text{concn}\right)+\beta_{\text{3}}\left(\text{diet}:\text{arsenic}\;\text{concn}\right)+\varepsilon$$

and

$$\text{model}\;2\;\text{:}\;\text{physiological}\;\text{parameter}=\beta_{0}+\beta_{\text{1}}\left(\text{diet}\right)+\beta_{2}\left(\text{arsenic}\;\text{concn}\right)+\beta_{\text{3}}\left(\text{diet}:\text{arsenic}\;\text{concn}\right)+\beta_{\text{3}}\left(\text{taxonomic}\;\text{abundance}\right)+\varepsilon$$

An ANOVA quantified which model best fit the data and in so doing determined if inclusion of taxonomic abundance in the model significantly improved the explanation of variation in host physiology. The false-discovery rate was controlled using the qvalue package.

### Data availability.

All sequencing data used in this study have been deposited in the Sequence Read Archive under accession no. PRJNA473851. The code associated with the analyses of these data is publicly available at https://github.com/chrisgaulke/zn_as_2018.
